# Effect of Elevated Temperature on Load-Bearing Capacity and Fatigue Life of Bolted Joints in CFRP Components

**DOI:** 10.3390/polym18020182

**Published:** 2026-01-09

**Authors:** Angelika Arkuszyńska, Marek Rośkowicz

**Affiliations:** Faculty of Mechatronics, Armament and Aerospace, Military University of Technology, 00-908 Warszawa, Poland; marek.roskowicz@wat.edu.pl

**Keywords:** CFRP mechanical joints, elevated temperature, load-bearing capacity, fatigue life, DSC testing

## Abstract

The search for innovative solutions in the field of construction materials used in aircraft manufacturing has led to the development of composite materials, particularly CFRP polymer composites. Composite airframe components, which are required to have high strength, are joined using mechanical fasteners. Considering that the composite consists of a polymer matrix, which is a material susceptible to rheological phenomena occurring rapidly at elevated temperature, there is a high probability of significant changes in the strength and performance properties. Coupled thermal and mechanical loads on composite material joints occur in everyday aircraft operation. Experimental tests were conducted using a quasi-isotropic CFRP on an epoxy resin matrix with aerospace certification. The assessment of changes in the strength parameters of the material itself showed a decrease of approx. 40% in its short-term strength at 80 °C compared to the ambient temperature and a decrease in the load-bearing capacity of single-lap bolted joints of over 25%. Even more rapid changes were observed when assessing the fatigue life of the joints assessed at ambient and elevated temperature. In addition, the actual glass transition temperature of the resin was determined using the DSC technique. Analysis of the damage mechanisms showed that at 80 °C, the main degradation mechanisms of the material are accelerated creep processes of the CFRP and softening of the matrix, increasing its susceptibility to damage in the joint area.

## 1. Introduction

CFRP (carbon fiber-reinforced polymer) composite materials are currently the primary material used in modern aircraft due to their high strength-to-weight ratio, corrosion resistance, and design flexibility, which allows for the design of lighter, more efficient, and durable aerospace structures. Carbon composites enable significant weight reduction, and thus also reduce fuel consumption and exhaust emissions [1,2,3,4]. Other advantages of CFRP materials include high specific strength and elastic modulus, excellent fatigue and fracture resistance, and corrosion resistance [1,2,3]. Manufacturing technology allows for the creation of complex shapes and large integrated components, supporting innovative aircraft designs [1,2]. New CFRP structures combine various functions, providing protection against lightning strikes, EMI shielding, and de-icing [5,6]. CFRP structures can be designed to ensure acoustic insulation and vibration damping [2,5].

Bolted joints are widely used to join CFRP components because they enable dependable and easily controllable joints. These characteristics are crucial in challenging applications such as the aerospace industry. Careful selection of bolted joint design parameters helps achieve significantly higher strength and stiffness [7,8,9]. Simplicity of inspection, maintenance, and replacement is critical for the safety of high-performance structures [7,10]. Bolted joints can maintain a prominent level of residual strength that allows continued safe use even after certain types of damage have occurred [10]. Optimization of the interference between the elements of a bolted node is possible by adjusting factors such as the assembly torque, the tolerance of the fastener hole, and the type of washer [7,11].

In everyday aircraft operation, composite airframe components are very often exposed to elevated temperature. Such conditions may occur during extended periods of aircraft standby on a sunlit airport apron. Exposure to sunlight—UV radiation and heat—can contribute to the degradation of polymers in aircraft structures. These factors cause the breakdown of polymer chains and reduce the mechanical strength of the material. An increase in the surface temperature of aircraft construction materials because of sunlight can lead to thermal expansion, softening, and accelerated chemical reactions [12,13,14]. Another example of aircraft exposure to elevated temperatures is high-speed flight. The aircraft is then exposed to intense aerodynamic friction between the air and its surface. This phenomenon can contribute to an increase in the temperature of the airframe, especially at the leading edges and thin aerodynamic profiles [15,16,17]. The surface temperature may also rise very rapidly locally at the point of lightning strike. This is due to the action of high-energy electric current and the Joule effect, which can cause local “hot spots” and potentially lead to structural damage [18,19,20]. In vertical take-off and landing (VTOL) aircraft, there is a risk of direct impact of hot exhaust gases on the airframe, especially during hovering and low-speed flight. The main cause here is the phenomenon of recirculation [21,22].

Exposure of composite aircraft airframes to elevated temperature poses a severe problem for structural integrity, durability, and safety. Elevated temperatures can adversely affect mechanical properties, especially when they approach or exceed the glass transition temperature (T_g_) of the resin [23,24,25]. Prolonged exposure to elevated temperature leads to degradation of the matrix and its bond with the reinforcing fibers. This can result in a loss of interlaminar shear strength and, consequently, a transition from brittle to dominant failure mode through complete delamination [26,27]. Elevated temperature significantly affects not only the CFRP composite material itself, but also the mechanical joints of the components made of it. Reduced load-bearing capacity and stiffness of the joints as well as accelerated damage propagation are observed. The increase in temperature contributes to a reduction in contact pressure and friction in the joint, which further reduces its efficiency [28,29,30]. Higher temperature intensifies the relaxation of preload forces in bolted joints. This reduces the effectiveness of the initial tightening and increases the risk of loosening the joint [31]. With increasing temperature, the damage propagation process and the time to failure are shortened. Although the macroscopic type of damage, which is usually bearing damage, remains unchanged, the speed and severity of damage increase, especially when moisture is present in addition to elevated temperatures [29,30]. Differences in the thermal expansion coefficients between composite components and metal mechanical fasteners can also cause significant thermal stresses [24,32].

One of the most popular epoxy resins used by many aircraft and glider manufacturers is L285 (MGS, Stuttgart, Germany), which obtained aerospace certification in 1985. It can be used for laminating carbon, glass, as well as aramid fibers. According to the manufacturer’s technical data sheet, heating at 50–55 °C allows the strength requirements for gliders and motor gliders to be met. For a component made with L285 resin to be successfully used in the construction of an engine-powered aircraft, it must be heated to 80 °C. In this case, the operating temperature range reaches 100 °C, and the glass transition temperature of the resin in combination with the H287 hardener is 90–95 °C [33].

While numerous studies have addressed the influence of elevated temperatures on the mechanical properties of CFRP composites, relatively little attention has been paid to the combined assessment of static strength, fatigue behavior and thermal transformations in mechanically fastened joints at temperatures close to the glass transition temperature of the polymer matrix. In particular, the interaction between temperature-dependent matrix softening, joint mechanics and fatigue damage evolution is still not sufficiently understood. The innovative nature of this study lies in the integrated experimental investigations of the temperature-dependent tensile strength of a quasi-isotropic CFRP laminate, the load-bearing capacity and fatigue life of single-lap CFRP bolted joints, and the determination of the actual glass transition temperature of the epoxy matrix using differential scanning calorimetry. Emphasis was placed on temperatures close to the glass transition range, which are relevant to actual operating conditions in aerospace applications. The results provide new insights into the degradation mechanisms of CFRP bolted joints subjected to combined thermal and mechanical loading.

## 2. Materials and Methods

### 2.1. Research Object

In the strength tests, a composite material was used, made from a carbon woven fabric with a basis weight of 160 g/m^2^ (KORDCARBON, Strážnice, Czech Republic) and an epoxy resin with aerospace certification L285 (MGS, Stuttgart, Germany) combined in a ratio of 100:40 with a slow-curing hardener 287 (MGS, Stuttgart, Germany). Thirteen fabric layers were stacked in a symmetric and balanced quasi-isotropic configuration with a 30° orientation increment, according to the following stacking sequence: [0°/30°/60°/90°/120°/150°/0°/150°/120°/90°/60°/30°/0°]. The composite was cured in two stages: first for 24 h at ambient temperature using a vacuum bag technique, and then at 80 °C in a laboratory oven (POL-EKO, Wodzisław Śląski, Poland). A composite material with a thickness of 2.5 ± 0.1 mm was obtained. The fiber volume fraction was estimated based on laminate mass, thickness, and constituent densities and was approximately V_f_ ≈ 64% (typical for vacuum-assisted hand lay-up aerospace laminates). Rectangular specimens with a width of 25 mm and a length of 300 mm were prepared for the tests.

**Figure 1:**
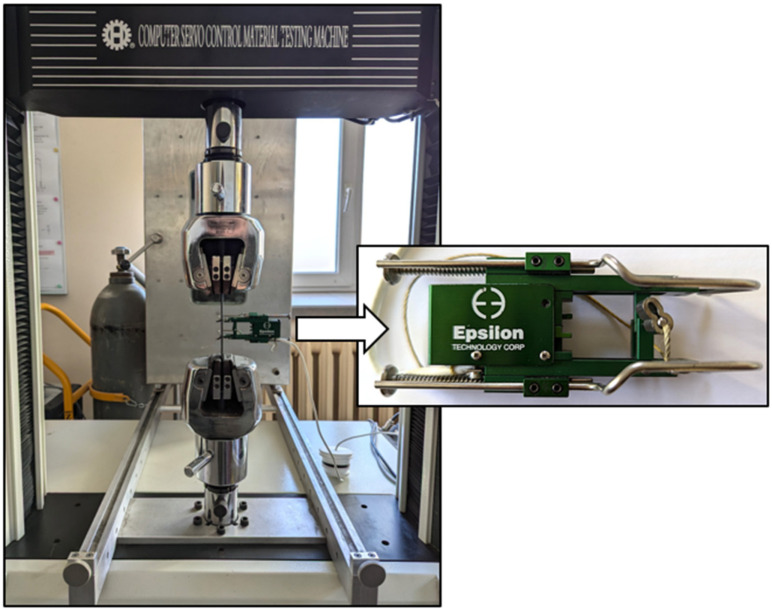
Composite specimen mounted in the jaws of the Hung Ta 2402 testing machine with the Epsilon extensometer.

Young’s modulus of the composite determined using an extensometer 3542-025M-025-HT2 (Epsilon, Jackson, FL, USA) with a measuring base of 25 mm and a universal testing machine Hung Ta 2402 (Hung Ta, Taichung City, China)—Figure 1, was equal to 38.75 GPa, while its tensile strength was 457 MPa.

**Figure 2:**
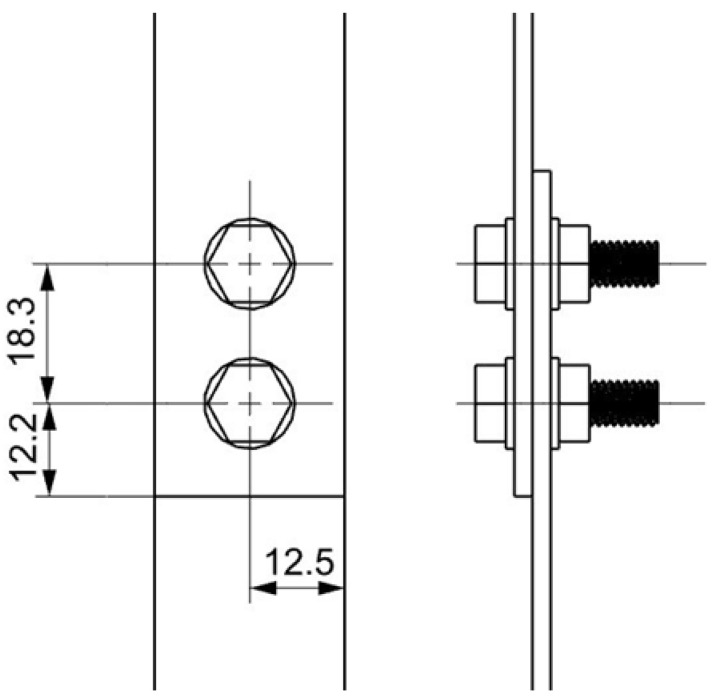
Scheme of the bolt node geometry.

For assessing the load-bearing capacity and fatigue life of mechanical joints, single-lap bolted joints of two composite elements were prepared. The geometry of the bolted joint is shown in Figure 2. M6 bolts made of steel with strength class 12.9 were used.

**Figure 3:**
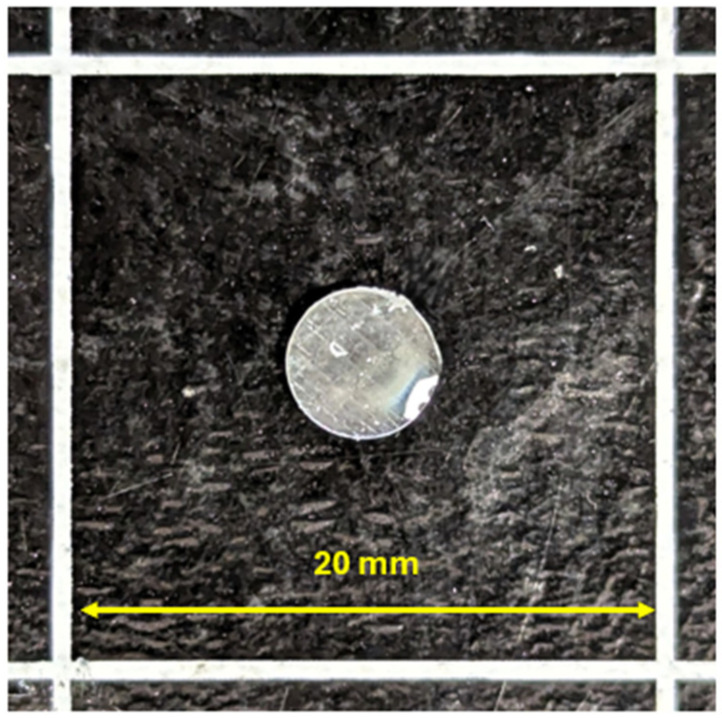
Epoxy resin specimen for determining the glass transition temperature.

To determine the glass transition temperature of the resin, it was necessary to prepare specimens in the form of discs with a diameter of 5 mm and a thickness of 1 mm—Figure 3. The average specimen weight was 21 mg. The resin preparation process was identical to that for the laminating resin—the ratio of L285 resin to H287 hardener was 100:40, and two-stage curing was conducted for 24 h at ambient temperature, followed by 6 h at 80 °C.

### 2.2. Mechanical Testing

**Figure 4:**
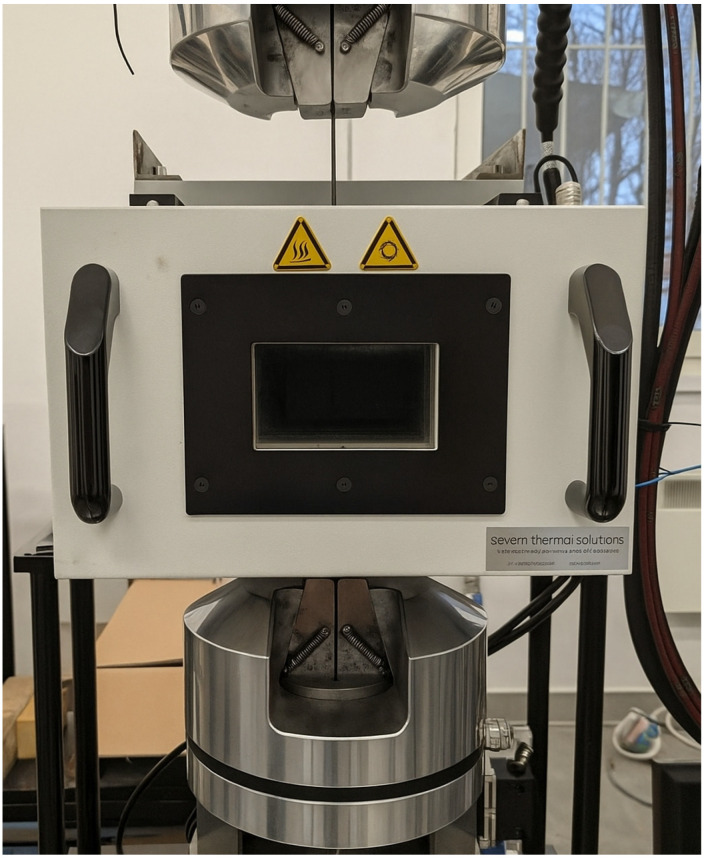
View of the specimen in the thermal chamber.

Static strength and fatigue life tests were conducted under controlled temperature conditions using a universal hydraulic testing machine MTS 809 Axial (MTS, Eden Prairie, MN, USA) with a Severn Thermal Solutions thermal chamber (Severn Thermal Solutions, Dursley, UK)—Figure 4. The tests were conducted at temperatures of 22 °C (ambient temperature), 40 °C, 60 °C and 80 °C. The deformation rate during the static tensile test was 2 mm/min, while the frequency during the fatigue life test was 5 Hz. This frequency was selected to avoid additional self-heating of the specimens, as confirmed by the stable temperature readings inside the thermal chamber during preliminary tests under ambient conditions.

The results of the static tensile test were used to determine the variable load ranges for the fatigue life test. The lower limit of the range was the same for all temperature values and was 4 kN for material specimens and 3 kN for bolted joints. The upper limit was approximately 70% of the static strength.

Prior to elevated temperature testing, all specimens were placed in a thermal chamber and preheated for 15 min, followed by temperature stabilization period of 30 min at the target temperature. This procedure ensured uniform thermal equilibrium throughout the CFRP material and bolted joint components, considering the low thermal conductivity of polymer composites. This procedure was applied to both material specimens and bolted joint specimens.

Three specimens were evaluated for each test configuration and temperature level.

### 2.3. Determination of the Glass Transition Temperature of the Resin

**Figure 5:**
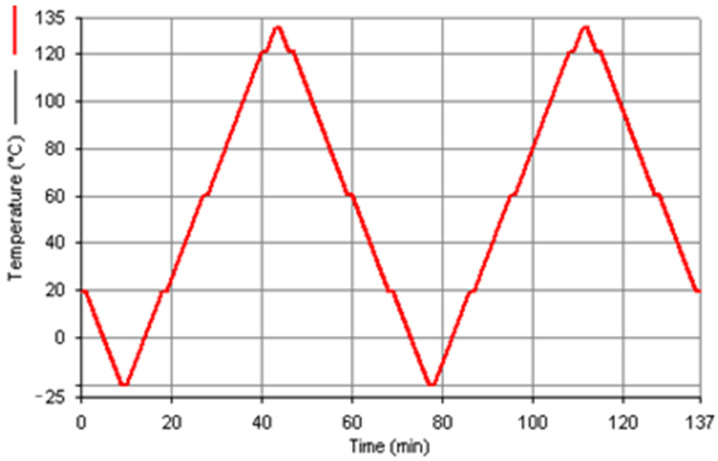
Temperature changes in the test program.

**Figure 6:**
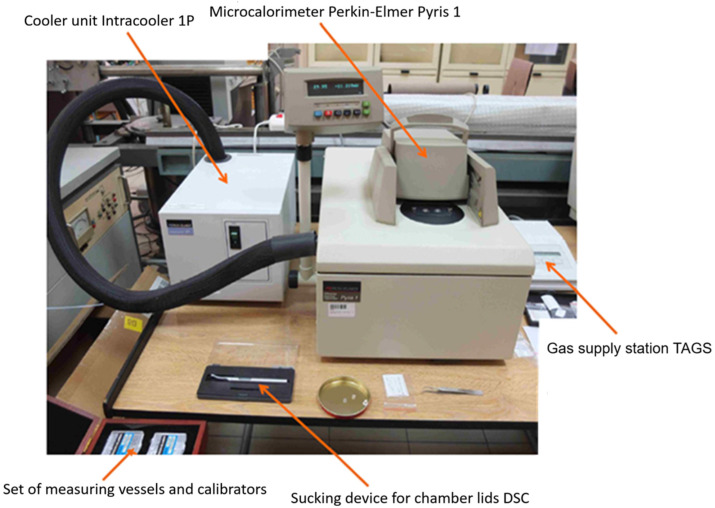
DSC measurement setup.

The actual glass transition temperature of the resin was determined using differential scanning calorimetry (DSC), a technique for measuring heat capacity, or more precisely, the change in heat flow between the test specimen and the reference specimen during thermal transformation [34,35]. A test program (Figure 5) was developed, consisting of heating and cooling the specimen twice from −20 to +130 °C. The temperature was changed at a rate of 5 K/min. The microcalorimetric measurement setup shown in Figure 6 consisted of the following main devices: a PerkinElmer Pyris 1 TGA scanning microcalorimeter with power compensation (PerkinElmer, Waltham, MA, USA), an Intracooler 1P chiller, a TAGS gas supply station, and a computer with software for measurement control and data analysis.

To achieve high measurement accuracy, an additional thermally inert reference material was used—sapphire, which has no changes in heat capacity and transformations in the tested range and has a well-known specific heat value. The apparatus was thermally calibrated by recording the signal for an empty specimen vessel. The purpose of this procedure is to eliminate effects related to the measuring system.

## 3. Results

**Figure 7:**
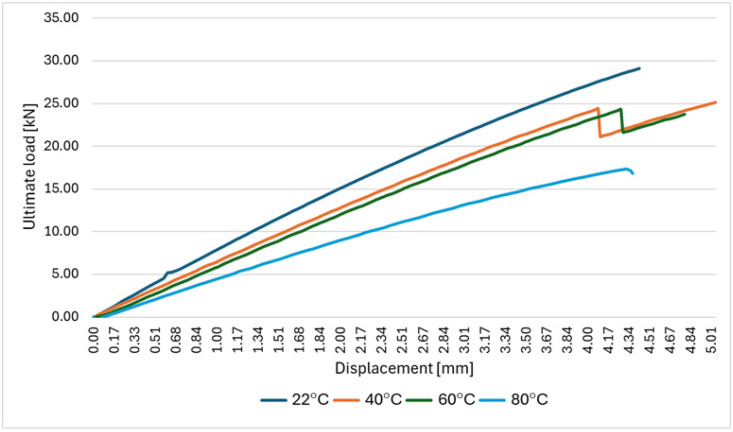
Force–displacement curves for tensile tests on CFRP specimens at different temperature levels.

Based on the data contained in Table 1 and the tensile curves shown in Figure 7, it was found that an increase in temperature leads to a significant decrease in the maximum load at which the CFRP composite material is damaged. The reference value is the average value of the force required to damage the composite at ambient temperature (approximately 22 °C)—29,153 N. An increase in temperature to 40 or 60° causes a slight decrease in the average maximum load—by several percent. However, a further increase in temperature up to 80 °C causes a sudden drop to an average value of 17,358 N.

**Figure 8:**
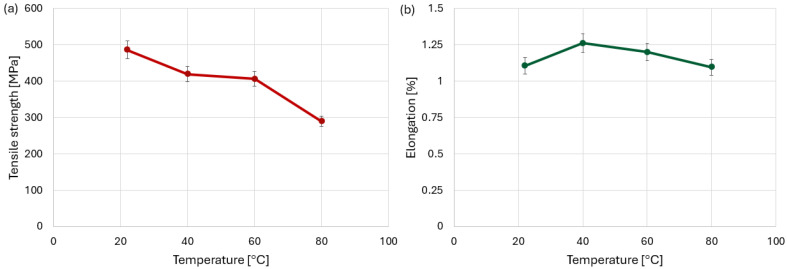
Graphs (**a**) tensile strength and (**b**) elongation of CFRP specimens at different temperature levels.

The corresponding average tensile strength values shown in Figure 8a decrease monotonically with increasing temperature. The highest average strength was obtained at room temperature (22 °C), while a reduction of approximately 40% was observed at 80 °C, which is consistent with progressive matrix softening as the temperature approaches the glass transition range of the epoxy resin.

The visible differences between the maximum displacement observed on the force–displacement curve (Figure 7) and the average elongation values summarized in Figure 8b result from the fundamental difference between the crosshead displacement and the specimen elongation. The displacement shown on the force–displacement curve corresponds to the total movement of the crosshead recorded by the testing machine. This value includes not only the deformation of the CFRP material but also additional factors affecting compliance, such as fixture and machine compliance, clearance, frictional slip, and preload relaxation.

Analysis of the elongation values of the test specimens at failure (Figure 8b) showed that an increase in temperature by 20–40 °C causes an increase in elongation. The maximum average elongation observed at 40 °C can be associated with a temporary increase in matrix ductility, where the material can accommodate higher strain before catastrophic damage initiates. At a temperature of 80 °C, however, the elongation reaches a value similar to that at room temperature.

In the case of single-lap bolted joints of CFRP elements, a decrease in load-bearing capacity with increasing temperature was also observed, but to a lower extent. The load-bearing capacity of the joint at 80 °C was 10,798 N, which was 25% lower than at 22 °C (14,546 N)—Table 2.

**Figure 9:**
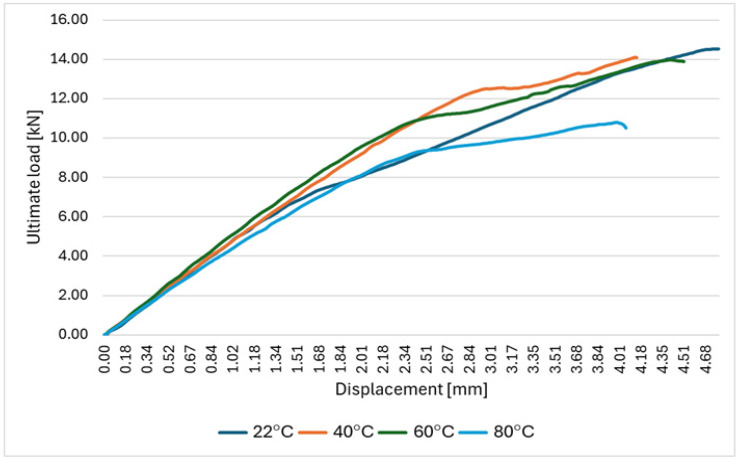
Force–displacement curves for tensile tests on bolted specimens at different temperature levels.

At elevated temperatures, partial softening of the epoxy matrix promotes increased local slip, bearing deformation, and accelerated viscoelastic creep in the joint region, resulting in higher crosshead displacement prior to failure (the largest at 60 °C)—Figure 9.

**Figure 10:**
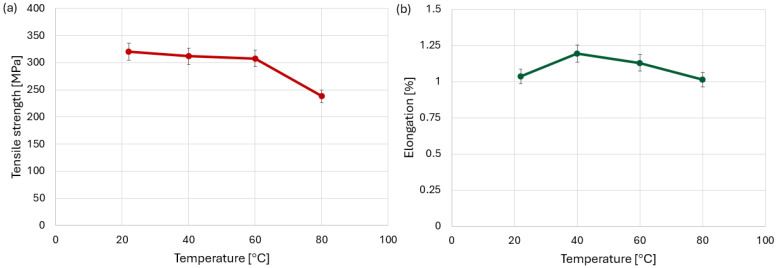
Graphs (**a**) tensile strength and (**b**) elongation of bolted specimens at different temperature levels.

However, this increased displacement does not directly correspond to higher average elongation (Figure 10b) of the joint. In contrast, at 40 °C, the joint exhibits a more stable deformation process, leading to the highest averaged elongation values despite lower total displacement observed in individual force–displacement curves. At 80 °C, the elongation again reached a value like that at ambient temperature. This behavior indicates the contribution of accelerated viscoelastic deformation and a shortened failure process. Figure 10a shows that, as opposed to material specimens, tensile strength does not change significantly up to a temperature of 60 °C, but drops rapidly at 80 °C.

As the temperature approaches the glass transition temperature of the epoxy matrix, softening of the matrix and accelerated creep processes reduce the load transfer efficiency between the fibres and the matrix. In bolted joints, this effect is further amplified by local contact stresses, frictional heating, and preload relaxation. The mismatch in thermal expansion coefficients between the CFRP laminate and the steel fasteners promotes additional interfacial stresses, leading to rapid damage accumulation and a significant reduction in tensile strength.

**Figure 11:**
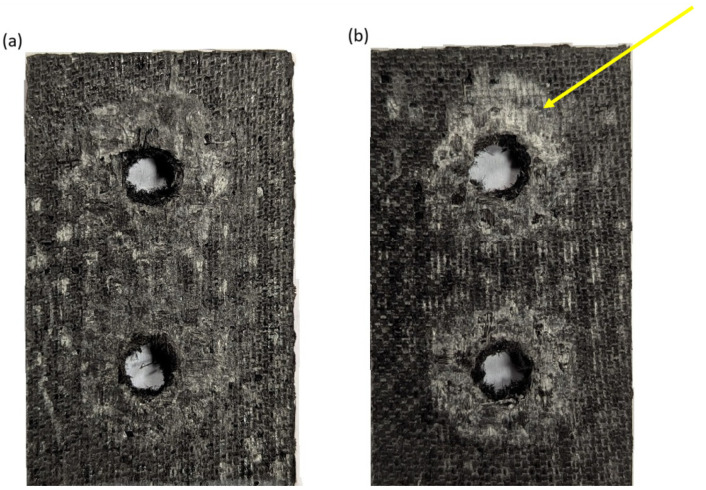
Comparison of disassembled CFRP components of the bolt node after strength testing (**a**) at a temperature of 22 °C and (**b**) at a temperature of 80 °C.

Figure 11 shows a visual comparison of damage to CFRP composite components that were part of a bolted joint subjected to tensile stress at two different temperature values. In the case of the specimen assessed at ambient temperature (Figure 11a), the area around the holes shows local damage, but the structure is mostly intact. The elevated temperature of 80 °C caused the damage to be significantly more extensive (Figure 11b). The area around the fasteners shows enlarged crushing and delamination areas. These effects are typical when the temperature approaches the glass transition point of the resin—the matrix softens significantly, increasing susceptibility to abrasion, deformation, and wear in the joint area.

**Figure 12:**
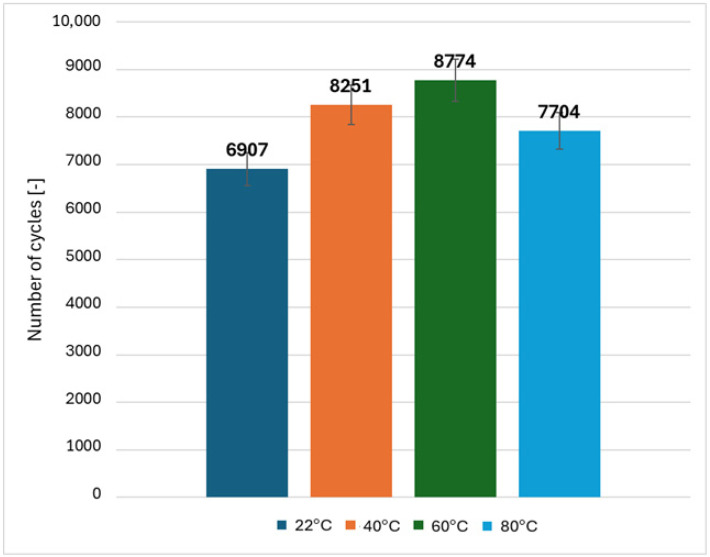
Comparison of average fatigue life of CFRP specimens at different temperature levels.

The average maximum load values listed in Table 1 and Table 2 were used to determine the variable load ranges in the fatigue life test—approximately 70% of the static strength was taken as the upper limit of the load range. Thus, the applied load varied in the range of 4–20 kN at 22 °C, 4–18 kN at 40 °C, 4–17 kN at 60 °C and 4–12 kN at 80 °C at a constant frequency of 5 Hz. This method of adjusting the load range resulted, as expected, in the fatigue life of the composite reaching the same order of magnitude for all temperature values—Figure 12. It results in similar stress ratios for each temperature condition.

**Figure 13:**
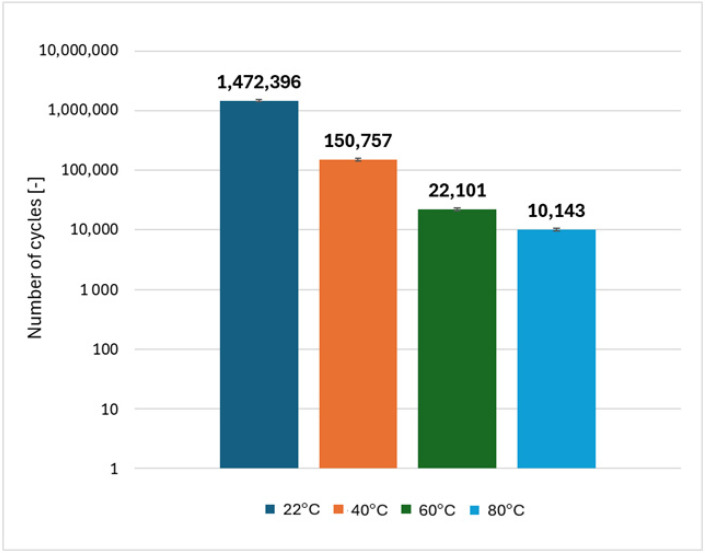
Comparison of average fatigue life of CFRP bolted joints at different temperature levels.

The application of the same method and the use of different load ranges for CFRP bolted joints (3–10 kN at 22 °C, 3–10 kN at 40 °C, 3–9 kN at 60 °C and 3–8 kN at 80 °C) did not provide consistent results. A further decrease in fatigue life was observed with increasing temperature. The fatigue life of the bolted joint at 80 °C, despite the significantly lower load conditions, is only 0.5% of the fatigue life of the joint at room temperature—Figure 13. This effect may be related to differences in thermophysical properties (different thermal expansion coefficients) between the CFRP composite and steel mechanical fasteners, as well as the cumulative effect of prolonged cyclic loading at elevated temperature.

The results indicate that the fatigue behavior of CFRP bolted joints is significantly more sensitive to temperature than that of the CFRP material itself. This pronounced degradation is associated with the temperature-dependent compliance of the joint system, which promotes earlier stiffness degradation and accelerates damage accumulation under cyclic loading. As a result, fatigue failure occurs after a reduced number of load cycles at elevated temperatures.

**Figure 14:**
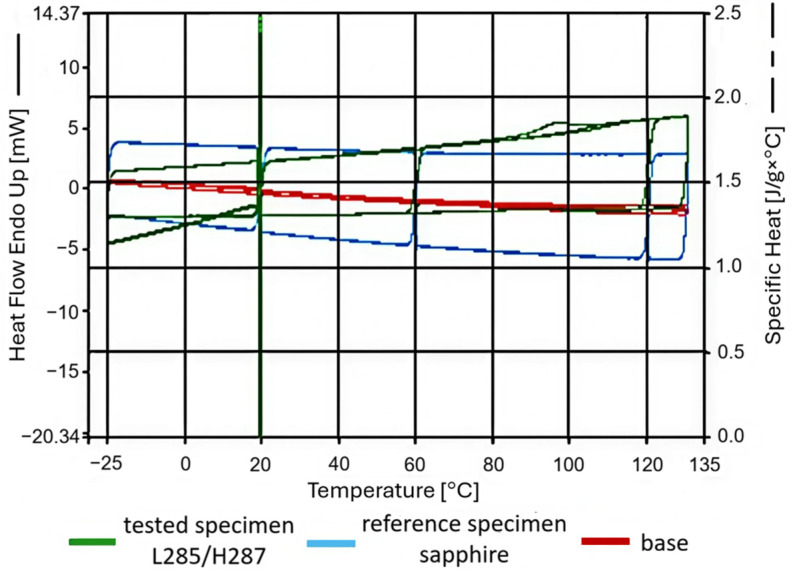
Example thermogram obtained.

The strength tests conducted so far have shown that the properties of CFRP composites change already at a temperature of 80 °C, which may indicate that the glass transition of the resin is approaching. The actual glass transition temperature was determined using differential scanning calorimetry (DSC). Figure 14 shows an example of a recorded thermogram, where the green color represents the test specimen made of L285/H287 resin, the blue color represents the sapphire reference specimen, and the red color represents the base—an empty vessel test.

**Figure 15:**
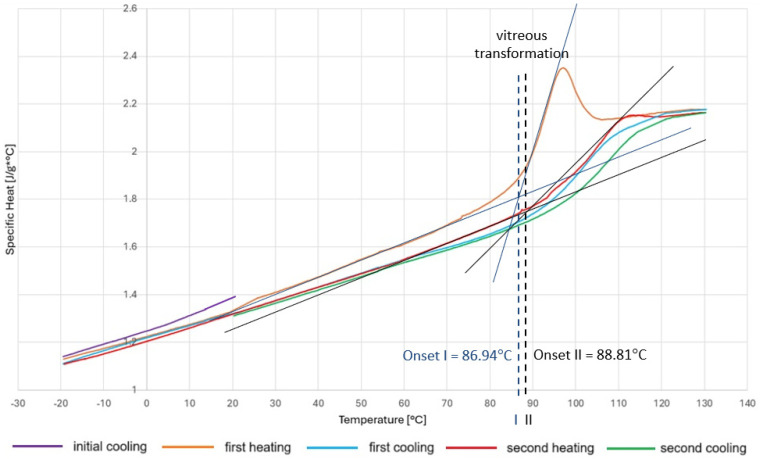
Graph of the specific heat of resin L285/H287 as a function of temperature with the glass transition marked.

The graph in Figure 15 shows the relationship between specific heat and temperature. The purple color represents the initial stage of cooling the specimen from ambient temperature to the initial temperature of −20 °C. The orange line represents the first stage of heating, during which there is a sudden jump at around 95 °C on the graph, which may indicate water desorption or residual polymerization. The second heating curve (in this case red) is more suitable for determining the glass transition temperature because it eliminates the influence of the material’s thermal history. No peak is observed here because the material has reached a stable state. The glass transition temperature of the resin was determined to be approximately 86 °C during the first heating and 88 °C during the second heating, using both the tangent intersection graphical method and dedicated analysis software.

The DSC results are consistent with the temperature range at which significant degradation of mechanical properties was observed during the strength tests. The glass transition temperature turned out to be slightly lower than that specified by the manufacturer, which should be considered, especially in a field as important for safety as aviation.

## 4. Conclusions

A key contribution of this study is the direct comparison between temperature-dependent behavior of CFRP material specimens and mechanically fastened joints manufactured from the same laminate system, allowing joint-specific degradation mechanisms to be clearly identified.

The strength tests confirmed the significant impact of elevated temperature on CFRP material and its joints. When the temperature rose from ambient temperature to 80 °C, the tensile strength of the composite decreased by 40%, while the load capacity of the bolted joints decreased by 25%.

Fatigue life tests showed that when the load range for the composite material itself was adjusted to its static strength at different temperature values, the same order of magnitude of fatigue life was achieved. This procedure was not effective for bolted joints—despite the reduction in the load range, a further decrease in fatigue life was observed. This is due to the proximity of materials with different thermal expansion coefficients and different heat capacities—CFRP and steel bolts, as well as longer exposure to elevated temperature.

A comparison of the damage mechanisms of specimens loaded at different temperature values showed increased damage due to friction in composite elements at a temperature of 80 °C. Softening of the composite matrix reduces abrasion resistance and increases susceptibility to deformation and wear.

The glass transition temperature of the L285/H287 resin used, determined by differential scanning calorimetry, is approximately 86–88 °C, which is lower than the manufacturer’s recommended range of use. It is therefore important to use appropriate safety factors, especially since the properties of the resin are significantly influenced by the preparation process. The results highlight the critical importance of considering thermal effects when designing bolted joints in CFRP components for aerospace applications.

This study is subject to certain limitations. The number of specimens evaluated was restricted to three for each configuration, and the results reflect short-term thermal exposure under controlled laboratory conditions. Future studies to further evaluate the durability of CFRP bolted joints under actual operating conditions should consider factors such as the effects of long-term thermal ageing, moisture absorption, and combined hygrothermal effects. Nevertheless, the consistent trends observed across all temperature levels clearly demonstrate the critical influence of temperatures approaching the glass transition temperature on the mechanical properties of CFRP bolted joints.

## Figures and Tables

**Table 1:** Ultimate load values and their decline depending on temperature for CFRP specimens.

Temperature [°C]	Ultimate Load [N]	Decline [%]
22	29,153	-
40	25,145	13.75
60	24,378	16.38
80	17,358	40.46

**Table 2:** Ultimate load values and their decline depending on temperature for bolted specimens.

Temperature [°C]	Ultimate Load [N]	Decline [%]
22	14,546	-
40	14,138	2.80
60	13,970	3.96
80	10,798	25.77

## Data Availability

The original contributions presented in this study are included in the article. Further inquiries can be directed to the corresponding author.

## Abbreviations

The following abbreviations are used in this manuscript:

CFRP	Carbon Fiber-Reinforced Polymer
DSC	Differential Scanning Calorimetry
EMI	Electromagnetic Interference
UV	Ultraviolet
TAGS	Thermal Analysis Gas Station
VTOL	Vertical Take-Off and Landing
